# Supplementary material for: Newly Discovered Alleles of the Tomato Antiflorigen Gene SELF PRUNING Provide a Range of Plant Compactness and Yield
Source: Int J Mol Sci. 2022 Jun 28;23(13):7149. doi: 10.3390/ijms23137149 (PMC9266710; doi:10.3390/ijms23137149)
Supplement: Supplementary file 1 [file ijms-23-07149-s001.zip › Supplementary material/Kang et al Suplementary materials.pdf]

Supplementary Materials

Newly discovered alleles of the tomato antiflorigen gene *SELF PRUNING* provide a range of plant compactness and yield

Min-Sung Kang[1,*], Yong Jun Kim[1,*], Jung Heo[1], Sujeevan Rajendran[1], Xingang Wang[2], Jong Hyang Bae[3], Zachary Lippman[2,4], and Soon Ju Park[1,†]

[1] Department of Biological Science and Institute of Basic Science, Wonkwang University, Iksan 54538, South Korea

[2] Cold Spring Harbor Laboratory, Cold Spring Harbor, NY 11724, USA
[3] Department of Horticulture Industry, Wonkwang University, Iksan 54538, South Korea
[4] Howard Hughes Medical Institute, Cold Spring Harbor Laboratory, Cold Spring Harbor, NY, USA

* M.K. and Y.J.K. contributed equally to this work.
† Correspondence should be addressed to S.J.P. (sjpark75@wku.ac.kr)

This PDF file includes

Figures S1 to S2

Legends of Tables S1 to S8

Figure S1

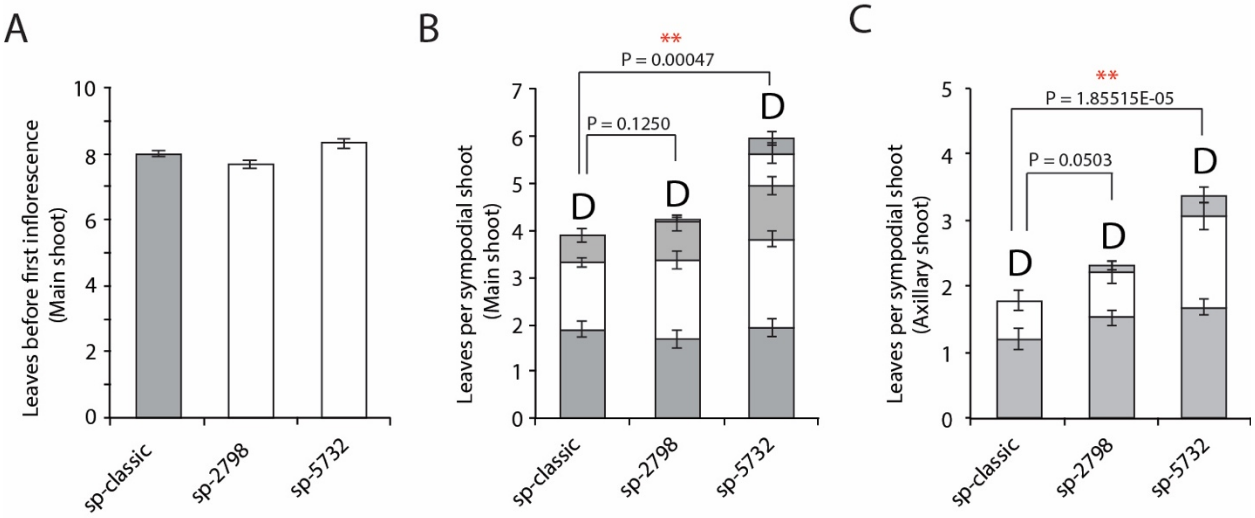

Supplementary Figure S1. Comparison of leaf numbers on primary and sympodial shoots among BC3F3 generations of *sp* alleles: (**A-C**). Quantification and comparison of primary-shoot flowering time (**A**) and sympodial-shoots initially produced by the primary shoot (**B**) and axillary shoot (**C**) in *sp* alleles. Statstic analyses were done with at least 16 biological replicates for each genotype. P values were determined via two-tailed, two-sample t-test; $^{**}P < 0.01$. CCs of *sp* alleles backcrossed with cv M82 more than four times.

Figure S2

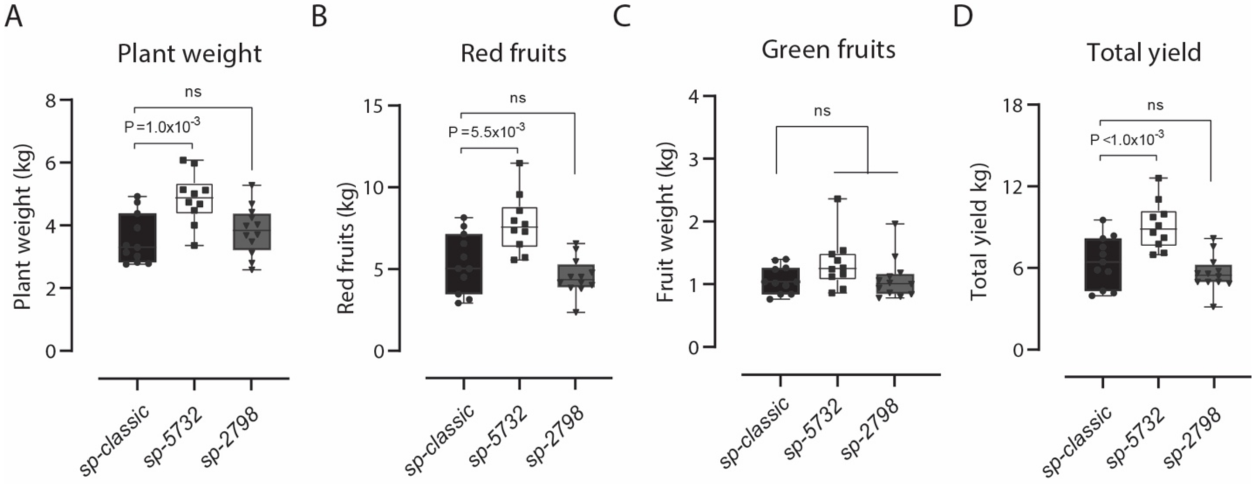

Supplementary Figure S2. Quantifications and comparisons of tomato yields among *sp* alleles at the second field trial:

(**A-D**). Statistical comparisons of mean values (±s.e.m.) for plant weight (**A**), red fruit weight (**B**), green fruit

weight (**D**), and total yield (**D**) from *sp-classic* as the control (black boxes), *sp-2798* (gray boxes), and *sp-5732* (white boxes). *P* values was determined via two-tailed, two-sample t-test; ns, no significant difference. Statistical comparisons were conducted with more than 10 biological replicates.

Table S1. List of Core Collection accessions selected by *sp-classic* genotyping marker

Table S2. Genotyping data of *sp* alleles using resequencing data of 588 accessions

Table S3. DEGs identified between *SP* and *sp-classic* in TM

Table S4. DEGs identified between *SP* and *sp-classic* in SYM

Table S5. DEGs identified between *SP* and *sp-classic* in both TM and SYM

Table S6. Enriched GO terms of total, TM, SYM, and single-/co-regulated DEGs

Table S7. Gene list categorized as developmental process and transcription enriched GO term analysis

Table S8. Information of used primers in this study
